# Non-Equilibrium Plasma Processing for the Preparation of Antibacterial Surfaces

**DOI:** 10.3390/ma9070515

**Published:** 2016-06-25

**Authors:** Eloisa Sardella, Fabio Palumbo, Giuseppe Camporeale, Pietro Favia

**Affiliations:** 1 Istituto di Nanotecnologia, Consiglio Nazionale delle Ricerche, Via Orabona 4, 70126 Bari, Italy; fabio.palumbo@cnr.it (F.P.); pietro.favia@uniba.it (P.F.); 2 Dipartimento di Chimica Università degli Studi di Bari “Aldo Moro”, Via Orabona 4, 70126 Bari, Italy

**Keywords:** antibacterial coatings, plasma processing, surface characterization

## Abstract

Non-equilibrium plasmas offer several strategies for developing antibacterial surfaces that are able to repel and/or to kill bacteria. Due to the variety of devices, implants, and materials in general, as well as of bacteria and applications, plasma assisted antibacterial strategies need to be tailored to each specific surface. Nano-composite coatings containing inorganic (metals and metal oxides) or organic (drugs and biomolecules) compounds can be deposited in one step, and used as drug delivery systems. On the other hand, functional coatings can be plasma-deposited and used to bind antibacterial molecules, for synthesizing surfaces with long lasting antibacterial activity. In addition, non-fouling coatings can be produced to inhibit the adhesion of bacteria and reduce the formation of biofilm. This paper reviews plasma-based strategies aimed to reduce bacterial attachment and proliferation on biomedical materials and devices, but also onto materials used in other fields. Most of the activities described have been developed in the lab of the authors.

## 1. Introduction

### 1.1. Materials Related Infections and Common Antibacterial Approaches

Bacterial adhesion on biotic and abiotic surfaces is a widespread problem in many fields, and its prevention has dictated considerable research efforts during the last decades. In healthcare, the function of therapeutic and diagnostic devices can be compromised by nonspecific adsorption of proteins and adhesion of cells onto device surfaces during long-term in vivo and ex vivo exposure to physiologic fluids. A good example is the case of implanted prosthesis: upon implantation, a competition exists between integration of the device into the surrounding tissue and adhesion of bacteria at the implant surface. For successful implants, tissue integration should occur before appreciable bacterial adhesion, thereby preventing colonization at the implant. However, host defenses are often unable to prevent bacteria adhesion and colonization [1]. In this case, bacteria first adhere to the biomaterial interface, then actively bind to the extra-cellular matrix (ECM) surrounding the implant, and form a protein layer at its surface. In this process, bacteria progressively form a biofilm, i.e., a well-defined metabolic state of bacteria life cycle where microbial cells, driven by a quorum sensing mechanism, lower their growth rate and baseline metabolism and express mechanisms leading to cellular aggregation in an amorphous polysaccharide matrix, also called slime. Biofilms allow bacteria to survive in harsh environmental conditions [2]. Nosocomial infections like those related to the use of central venous catheters, urinary catheters, prosthetic heart valves, and orthopedic devices are clearly associated with biofilms adhering to biomaterial surfaces. Since bacteria in biofilms can evade host defenses and withstand antimicrobial systemic chemotherapy [3], the utility of antimicrobial coatings able to repel and/or kill bacteria appears clear.

Bacterial contamination is also a serious concern in food packaging and in several fields of industrial engineering. Antibacterial packaging materials are needed to control the microbial contamination of solid/semi-solid food by inhibiting the growth of microorganisms at its surface, which normally comes into direct contact with the packaging material. Controlling bacterial proliferation means, in the food industry, prolonging the shelf life of food without altering its organoleptic properties. It also prevents humans from being infected by food-borne diseases, mainly caused by microbiological spoilage and contamination of food with pathogen microorganisms [4,5]. It is of paramount importance that this target is achieved with materials well tolerated by the human metabolic system, with no use of allergenic or potentially toxic compounds. Since active packaging could potentially be applied to most foods, the audience interested in this technology is huge.

One of the most serious concerns in Industrial Engineering is the occurrence of biofouling and biofilms on surfaces in contact with proteins and other biologic contaminants. Biofouling is defined as the settlement and accumulation of floating organisms onto an inanimate substrate; marine biofouling is also a concern for skull and propellers of vessels [6]. In industrial settings, biofouling can often degenerate into bacterial biofilms, representing both a hygienic and an economic problem: they can contaminate the conveyor belts in the production process, reduce heat transfer and operating efficiency in heat exchange equipment, increase energy and water consumption, cause mechanical blockages and accelerate corrosion of metal surfaces [7]. Marine biofouling is also a concern for skulls and propellers of vessels.

Considering the high number of fields where surface bacterial contamination represents a severe danger, and the size of the related business, it is easy to understand why in the last decades a huge scientific and technological effort has been devoted to optimize and set up methods to prevent bacterial adhesion and proliferation on material surfaces. Accordingly, research has focused on the development of antibacterial surfaces and thin coatings that can be applied to different devices to confer resistance to bacterial colonization without affecting other properties. For instance, the visual clarity of contact lenses should not be affected by an antibacterial coating, and the flexibility of a vascular graft should not be limited by a bioactive coating on its lumen. In general, such coatings should not establish adverse effects on host bodies, cells and tissues. Moreover, given the variety of biomedical devices and implants, as well as bacteria, a single successful approach is not feasible, so antibacterial strategies need to be tailored to specific needs [8]. For contact lenses, for example, non-fouling coatings seem appropriate, as they will resist both the attachment of bacteria and biofouling [9]; on the other hand, for hip and knee prostheses, one wishes to deter bacteria for long periods of time, in order to avoid late-stage infections, while encouraging a good integration of the implant with human tissue. To achieve this target, a composite coating should be deposited at the implant surface, with the task of releasing antibacterial compounds in sub-inhibiting concentrations, and maintaining environmental drug concentrations higher than the Minimum Inhibiting Concentration (MIC) of the target organisms for a long time. This result can be achieved embedding bioactive compounds in polymeric matrices that limit their diffusion in the surrounding medium [10]. A fine level of control on the release kinetics could be acquired with hydrogel coatings, for example, from poly(ethylene glycol) di-acrylate and co-polymers; water absorption swells the polymer network and opens the way to the diffusion of the active compound [11,12]. Another common strategy for drug delivery is based on the biodegradability of the coatings: bioresorbable polymers, such as poly(lactic acid), can be degraded in newly formed human tissues into molecules that can be easily metabolized by human cells. Concurrent release of the active embedded compounds can take place while the coating is dissolved [13].

Drop casting/dip coating [1,14,15], Layer-by-Layer (LbL) deposition [16,17,18,19], sol-gel [20,21] and electrochemical deposition [11,12,22,23,24] are probably the most common ways for coating the surface of solid materials with a drug-delivering matrix providing a time-controlled release of bioactive ingredients (Table 1). Some processes are instead based on the covalent attachment of carboxylic, amine or thiol groups normally present in antibacterial molecules to proper surface reactive chemical groups exposed by functionalized polymeric coatings (see Table 1). Covalent immobilization has found a wide application in all technological fields requiring the permanence of the active compound on the topmost surface of the coatings, such as permanent bone prosthesis, industrial parts, nosocomial tools, etc. On the other hand, since the active ingredients are not released, these bio-conjugated surfaces cannot be effective through wide spatial ranges [25,26,27].

### 1.2. Plasma Technology

Plasma, the “fourth state of matter”, the most common state in nature, is ionized gas with equal density of positive and negative particles [31]. Thermonuclear plasmas apart, less energetic thermal (10^3^–10^4^ K) and cold (room temperature, RT) plasmas, populated by electrons, ions, atoms and molecules in different states, are relevant for technological applications. From their start in Microelectronics (Integrated Circuits, IC) and Photovoltaics (Solar Cells) in the 1970s, cold plasmas permeate several other industrial fields today, including the biomedical ones. Ref. [32] offers a brief Essay on the temporal evolution of plasmas in Science and Technology.

Cold (non-equilibrium) plasma discharges can be ignited at low (1–100 Pa, LP) or atmospheric pressure (AP) by applying a proper electrical field (generally in kHz, MHz or MW) to a gas/vapor mixture in properly configured plasma reactors/source. In a typical LP system, the “parallel plate” plasma reactor, the discharges is ignited between two parallel electrodes a few cm apart, with substrates positioned on one of the two. Many different configurations exist (electrodes, coils, movable substrates, etc.) to optimize plasma processes on particular substrates (flat, porous, webs, inside of narrow tubes, particles, etc.). AP systems include Dielectric Barrier Discharges (DBD) and AP plasma jets (APPJ). In DBDs, a dielectric layer (e.g., alumina) onto one or both electrodes, allow homogeneous (glow) rather than non-homogeneous (filamentary) discharges, and reduce the current (heating) through the system. An inter-electrode gap of a few mm is typical of DBD systems. In APPJs, the plasma is generated in a small caliber dielectric tube (e.g., with a metal needle electrode connected to the HV generator and a ground ring electrode wrapped around the tube), and then ejected on the substrate by the flow of the feed. Plasma reactors and sources can be precisely designed and optimized for LP and AP processes for processing small lab scale samples, or large scale high throughput industrial products [33].

By properly varying plasma parameters such as LP/AP regime, nature of the feed, reactor features and materials, frequency power and modulation of the electric field, and others, it is possible to properly tune density and distribution of the active species (atoms, radicals, ions, and electrons) generated by the fragmentation of the gas feed that interact with the exposed substrates. In this way, surface composition and properties of material substrates can be tailored in many possible ways with heterogeneous gas/solid reactions. Advantages of plasmas for materials processing include: the ability to modify thin surface layers of conventional materials with no alteration of the bulk, limited use of chemicals, dry technology with no use of solvents, easy integration in industrial processes, and intrinsic sterility. Three kinds of surface modification plasma processes can be defined, in general, as follows.

Dry etching: When defined species are generated in the plasma in the presence of certain materials (e.g., oxygen atoms vs. polymers, etc.), ablation reactions can occur, which form volatile species from the substrate (e.g., H_2_O and CO_2_ from polymers). By coupling cold (mostly LP) plasmas with micro/nano lithographic techniques, etching processes can be performed very precisely (e.g., on Si and other semiconductors), with lateral resolution of tens of nm and very high aspect ratio, to satisfy the needs of extreme miniaturization and newer materials for IC fabrication [34].

Plasma Enhanced Chemical Vapor Deposition (PE-CVD): Radical species are generated in LP/AP PE-CVD plasmas fed with proper chemicals to deposit coatings 1–10^3^ nm thick. Depending on the feed and on the experimental conditions, substrates can be decorated with coatings of many possible chemical compositions, including silica-like, silicone-like, fluorocarbon, diamond-like and others, for a wide range of properties (hardness, hydrophilic/phobic character, cytocompatibility, resistance to corrosion, etc.) that can be imparted at the surface of substrates. Since the chemical compound of the feed usually generates many possible fragments, building blocks of the coating, also volatile compounds without the functional groups (e.g., double bonds) needed for conventional polymerizations (e.g., methane), can originate PE-CVD coatings, whose stoichiometry can be varied in continuo with the plasma parameters. When organic compounds are used, the terms monomer for the feed and Plasma Polymerization for the technology are used.

Plasma Treatments: In these processes, materials are exposed to cold plasmas fed with reactive (e.g., O_2_, N_2_, H_2_, NH_3_, H_2_O vapor, N_2_/H_2_, etc.) or inert (Ar, He, etc.) non-polymerizable gases. Surface modifications imparted with Plasma Treatments are extremely shallow, a few nm, and include removal of surface contaminants, surface oxidation, and surface grafting of polar groups onto polymers to impart hydrophilic character, printability, affinity with other materials, anchor groups for (bio)molecules, cytocompatibility and other properties.

Nowadays, Plasma Sciences and Technology impacts three different areas of Medicine and Biology, namely:

- Plasma Medicine, i.e., the therapeutic use of cold AP air plasmas on living tissues for non-invasive surgery, wound sterilization and healing, blood clotting, teeth bleaching, cancer treatments, and other applications [35].
- Sterilization and decontamination of materials and devices [36].
- Surface modification of biomaterials, Tissue Engineering scaffolds, biosensors and medical devices [37] aimed to optimize the response of biological entities (proteins, bacteria, cells, fluids, and tissues) in contact with the modified material and drive, and, consequently, their behavior in vitro and in vivo.

This paper describes LP/AP non-equilibrium plasma-processes developed at the lab of the authors, aimed to develop antibacterial surfaces, namely: non-fouling Poly(Ethylene Oxide) (PEO)-like coatings; surface-immobilized antibacterial molecules; nano/bio-composite coatings releasing antibacterial biomolecules; and nano-composite coatings releasing antibacterial metal ions.

## 2. Non-Fouling Plasma Deposited Coatings

All surfaces allow, in principle, for the adhesion of several different species. Bacterial colonization of materials, in particular, is a surface-mediated process, that poses challenges to biomaterial scientists. A promising alternative to a conventional systemic therapy against infections is represented by engineered surfaces with tailored properties aimed at locally inhibiting bacterial adhesion and proliferation on medical devices. Fouling is a main concern in the case of surfaces exposed to aqueous environments where microorganisms can bind to a surface and form a conditioning layer, which then provides an easily accessible platform for other undesired species to attach and proliferate [38]. Hydrophobic/superhydrophobic surfaces have been proposed as anti bacterial surfaces, and they can be easily fabricated by means of PE-CVD [39]. Since the first conditioning layer of proteins and biomolecules would adhere through their hydrophobic moieties with weaker (e.g., Van der Waals) forces with respect to H-bonds and other forces acting through their hydrophilic (e.g., -COOH, -NH_2_, -OH, etc.) moieties, weaker adhesion of biofilms and bacteria and cells would result at these surfaces [40].

Hydrophilic polyethylene glycol (PEG) polymers, also referred to as polyethylene oxide (PEO), have been shown to resist to protein and cell (including bacteria) attachment [41]. Such surfaces are defined as “non-fouling” or “anti-fouling” [42,43,44]. This property is believed to correlate strongly with the hydration layer at the PEO surface [45], attributed to the presence of hydrophilic ether (CH_2_-CH_2_-O)_n_. functionalities. These groups create a water-solvated structure which forms a liquid-like surface with highly mobile disordered molecular chains [46,47,48]. For protein adsorption to occur, there must be a reduction in the dehydration entropic energy associated with the removal of surface bound water [45]. Due to this effect, the tightly bound water molecules entrapped in the PEO surface through hydrogen bonds form a physical and energetic barrier that cannot be displaced by proteins and cells. The unique non-fouling properties of PEO-modified surfaces have encouraged the development of several approaches (i.e., covalent immobilization, physical adsorption, self-assembled monolayers and plasma deposition) to PEO-like thin films [44,49,50,51,52,53,54,55,56,57,58].

Kingshott et al. [59] showed that physisorbed PEO polymers do not provide long-lasting reduction in bacterial adhesion, whereas PEO chains covalently attached to a substrate are effective. About these findings Vasilev et al. [8] hypothesize that bacteria act as “mega-surfactants” with high interfacial affinity for the material surface, displacing physisorbed polymer chains from the material surface, whereas covalently surface-grafted polymer chains resist such displacement.

Compared to other methods, LP PE-CVD processes from monomers with CH_2_CH_2_O moieties in their structure have been widely applied as a versatile tool to impart non-fouling properties with PEO-like coatings on a large variety of substrates [44,49,50,51,53,55,56,57,58,60]. Three important features have to be achieved for these coatings: good adhesion to substrates, stability in water media, and high retention of the CH_2_CH_2_O functionalities of the monomer. This latter parameter is known as *PEO character*, and can be evaluated by measuring the relative importance of the ether carbon component C1 at ~286.5 eV of Binding Energy in the C1s X-ray Photoelectron Spectroscopy (XPS) spectrum of the coating [49,50]. High PEO character is desirable in non-fouling surfaces for proteins and cells; coatings with low PEO character, instead, promote protein and cell/bacteria adhesion and proliferation [53]. Pre-treatments in LP plasmas fed with Ar can be useful to improve the adhesion of the PE-CVD coatings; the positive-ion bombardment associated to such pre-treatments cleans the substrates from contaminations and generates nano-roughening at their surface.

We have set-up a robust LP PE-CVD process to deposit PEO-like coatings on metals, ceramics and substrates such as polystyrene (PS), polyethylene terephthalate (PET) and glass, and studied how to deposit films with high PEO character [49,50,53]. PEO-like coatings were deposited from a feed of di-ethylene glycol dimethyl ether (DEGDME) vapors and Ar at constant flow rate ratio (0.40 sccm DEGDME, 5 sccm Ar) and pressure (400 mTorr). A stainless steel plasma reactor equipped with two “parallel plate” asymmetric stainless steel electrodes was used; the small (Ø 8 cm) one was connected to a radiofrequency (RF, 13.56 MHz) generator through a matching network while the large (Ø 18 cm) electrode was connected to ground. Varying the RF power input from 5 to 15 W allowed the deposition of coatings with different chemical compositions. A high degree of monomer fragmentation was achieved in the plasma at 15 W, resulting in coatings with a PEO character of about 40% and advancing/receding water contact angles (WCA) of 71° ± 5° and 50° ± 5°, respectively. At 5 W, coatings with a PEO character as high as 80% and more hydrophilic behavior (WCA_adv_ 56° ± 5°, WCA_rec_ 37° ± 5°) were obtained [53]. To confirm that the PEO character is correlated to the fouling/non-fouling behavior of the coatings, PE-CVD coated quartz crystals were exposed to a fibronectin (a cell adhesion protein) solution into a Quartz Crystal Microbalance (QCM-D) at 37 °C. The coatings deposited at 5 W, with high PEO-character, proved to be very stable in water solution, well adherent to substrates, with no leach of compounds and no protein adsorption from the solution. The adhesion of fibronectin was observed, instead, on coatings with lower PEO-character. The non-fouling activity of PEO-like coatings deposited at low power was confirmed by their low affinity with human telomerase cancer cells (hTERT-BJ1), keratinocytes and murine fibroblasts; cells could neither spread nor even adhere on PEO-like surfaces characterized by high PEO character [50,53].

Standing the proved non-fouling activity of PEO-like coatings plasma-deposited in mild LP plasma conditions, only few applications have been reported in literature about their use to prevent surface bacterial colonization [61,62,63]. The principles ruling the non-fouling properties of hydrophilic surfaces towards proteins and eukaryotic cells should also be valid for bacteria; PEO-like coatings LP plasma deposited from DEGDME should thus be able to inhibit protein and cell adhesion to solid substrates, as well as bacterial fouling. LP PE-CVD has proven to be a robust, versatile tool to produce this kind of surfaces and to adapt their characteristics to the application needs. Sardella et al. have plasma-deposited micro-patterned surfaces alternating PEO-like domains with cell adhesive ones derived from PE-CVD of acrylic acid. These coatings can drive cells to adhere only to specific regions of the substrates, leaving the non-fouling ones clean [50,53]. By combining PE-CVD with colloidal lithography, non-fouling PEO-like coatings could be generated on surfaces characterized by nanometric reliefs, that quenched the cell-adhesive properties of the rough PET substrate underneath [49].

AP PE-CVD processes have also received great attention for the deposition of PEO-like coatings [64]. Recently, the possibility to feed AP plasma processes with aerosols of liquids, of solutions or suspensions (e.g., of biomolecules or of nanoparticles, NPs) has been explored, with very interesting results. This approach allows the use of precursors with high boiling points that could not be used in LP processes or with common bubbling systems. We have studied how to deposit PEO-like films by means of aerosol-assisted AP plasma processes. A He aerosol of tetraethylene glycol dimethyl ether (TEGDME) was used as feed. Two different He flow rates were used: a constant He flow to generate the TEGDME/He aerosol with a constant flow of monomer, and a variable flow of He (carrier) to transport the aerosol. The effects of several experimental conditions on the chemical composition of the coatings were investigated, ranging from PEO-like non-fouling to cell-adhesive. Experiments were carried out in a parallel plate electrode DBD reactor. Each electrode (50 × 50 mm^2^; 4 mm gap) was covered with a thin alumina plate. The effects of applied voltage, excitation frequency (both influencing the power of plasma) and monomer dilution could be investigated on the PEO character and on the deposition rate of the coatings. Indeed, the AP regime allowed higher deposition rates compared to corresponding LP processes. The deposition rate could be improved by increasing the applied voltage (but the PEO-character of the coating became lower); increasing the frequency led both to a lower deposition rate and to a lower PEO-character. Increasing frequency and voltage, in fact, increases the plasma power and, as in LP processes, leads to higher monomer fragmentation and lower PEO-character. A significant reduction of intact ether moieties was also observed by increasing the flow of He carrier, which lowers the concentration of the aerosol and increases the monomer fragmentation [65,66].

## 3. Bio-Conjugated Plasma Modified Surfaces

**Figure 1:**
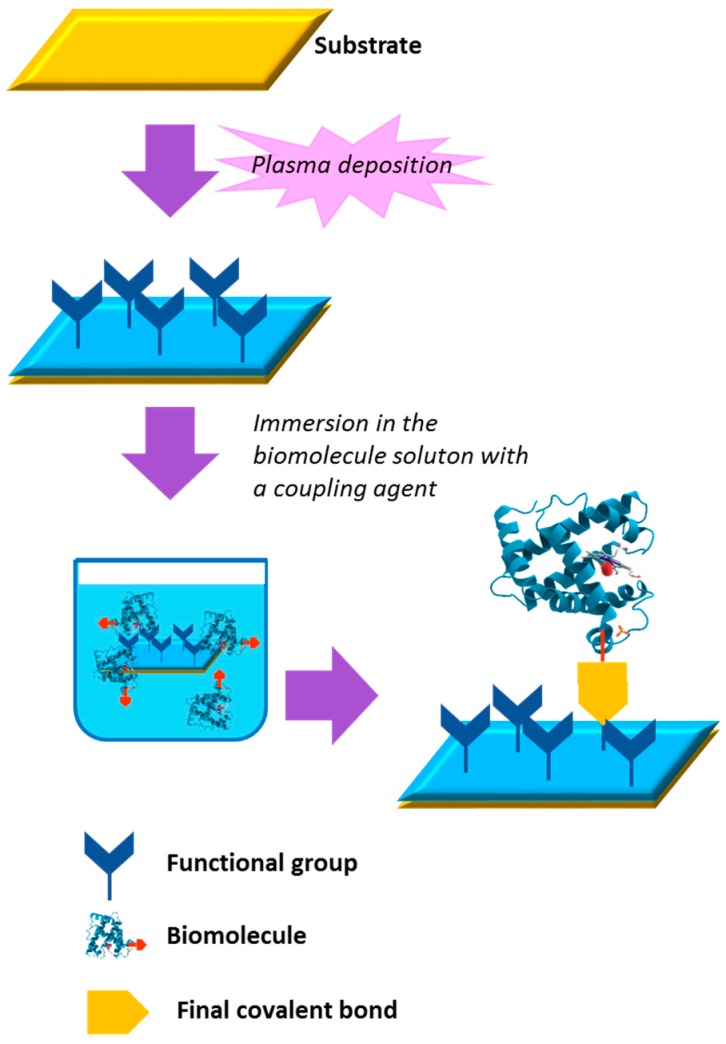
General scheme of a covalent immobilization on a functionalized plasma deposited coating.

Covalent grafting of antibacterial compounds onto biomaterial surfaces has been the subject of considerable research in the quest for antibacterial surfaces with long-lasting efficacy. Certain PE-CVD coatings and plasma treated surfaces are well suited as adhesive interlayers for the covalent surface immobilization of antimicrobial molecules for several reasons: ease of deposition; good adherence on most materials; presence of reactive chemical groups for covalent bonding not available on the material underneath. These processes, traditionally carried out in LP plasmas, usually include two main steps, as shown in Figure 1: an initial phase aimed at enriching the topmost surface of the substrate with functional groups (grafting oxygen- or nitrogen-containing reactive groups or depositing films functionalized with the same groups); adsorption or binding (e.g., by covalent bonds) of the bioactive molecule to the surface. Plasma-synthesized surfaces rich in amine, carboxylic, epoxy and aldehyde groups have been used by many scientists for their compatibility with well-established chemical reactions for immobilizing bioactive molecules such peptides, proteins, quaternary ammonium compounds, antibiotics, etc. [8,64,67,68,69,70,71].

Carboxyl-functionalized coatings deposited in LP plasmas have been exploited in our lab for the covalent surface attachment of bovine lactoferrin (BLF) and lactoferricin B (LfcinB), one of the sub-unities resulting from BLF digestion with pepsin. BLF and LfcinB are classified as antimicrobial peptides (AMPs), i.e., small cationic polypeptides (<10 kDa; 3–50 amino acid residues) produced by all organisms, from plants to insects to humans as a major part of their non-specific defenses against infections. They have good activity against most bacteria, and excellent activity (MIC of 1–4 μg/mL) against multidrug resistant *Pseudomonas aeruginosa* or methicillin-resistant *Staphylococcus aureus* [72]. The antimicrobial activity of BLF and LfcinB, immobilized on the -COOH groups of a PE-CVD ethylene/acrylic acid (pdEthAA) film, was evaluated to control *Pseudomonas* strains responsible for the spoilage of mozzarella cheese [68]. This procedure was already successfully tested for the immobilization of other biomolecules or bio-structures, such as vesicles, heparin and RDG cell-adhesive peptides [73,74,75,76,77]. Plasma deposition of pdEthAA films was carried out in a RF plasma reactor equipped with two parallel internal and symmetric stainless steel electrodes. The upper electrode is ground-shielded and connected to a RF (13.56 MHz) generator through a matching network, whereas the lower, sample holding electrode is connected to the ground. Using mild enough experimental conditions (i.e., 30 W) and a constant 3:1 flow ratio of ethylene and acrylic acid vapors, COOH-functionalized coatings highly stable in water could be deposited in polypropylene microtubes for bacterial culture. After PE-CVD, carboxylic groups were activated with 1-Ethyl-3-[3-dimethylaminopropyl] carbodiimide hydrochloride (EDC) and incubated with BLF or LficinB solutions to allow the covalent immobilization of both peptides in their active form, as confirmed by the reduction of the growth rate of milk spoilage bacteria *P. gessardii* and *P. fragi* after 24 h of cultivation in the modified microtubes [68].

**Figure 2:**
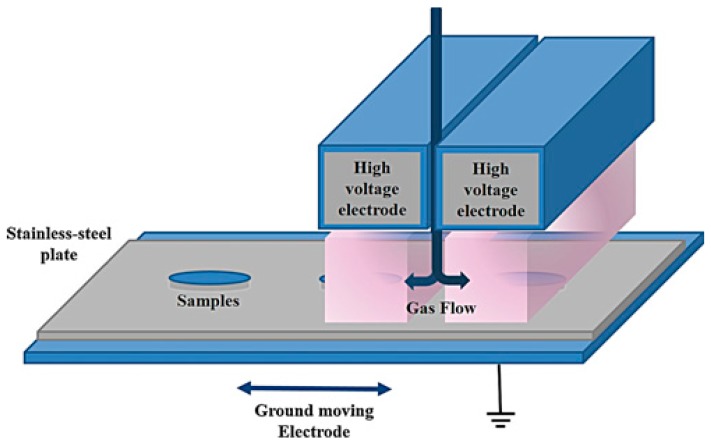
Schematic illustration of the AP-DBD source used for the PE-CVD from GMA, obtained from [69], with permission from © 2015 John Wiley and Sons.

**Figure 3:**
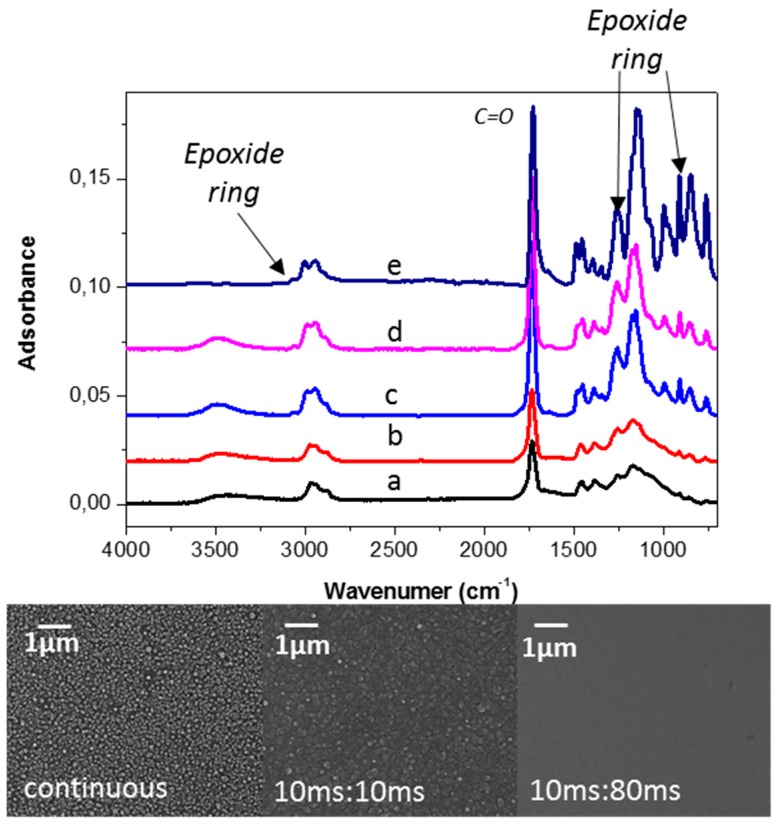
(**Top**) FT-IR spectra of PE-CVD GMA films deposited at 50 W at different on/off time ratio: (**a**) continuous; (**b**) 10 ms:10 ms; (**c**) 10 ms:40 ms; (**d**) 10 ms:80 ms; and (**e**) conventional GMA polymer. (**Bottom**) SEM images of PE-CVD GMA films deposited at 50 W at different on/off time ratio. Adapted from [69], with permission from © 2015 John Wiley and Sons.

**Figure 4:**
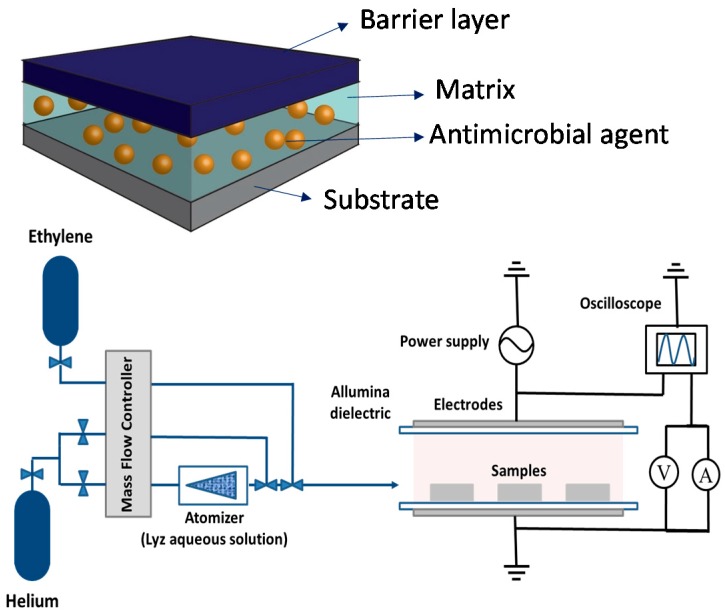
Sketch of the antibacterial containing nano/bio-composite coating (**top**) and schematic diagram of aerosol-assisted atmospheric-pressure DBD deposition system (**bottom**). Adapted from [82], with permission from © 2015 John Wiley and Sons.

In another approach, glycidyl methacrylate (GMA) was plasma-deposited in an AP-DBD to demonstrate that epoxy–containing interlayers can be effectively exploited for immobilizing Dispersin B (DspB), a 42 KDa protein with anti-biofouling activity [69]. The DBD source, shown in Figure 2, consisted of two high-voltage electrodes (1 mm gap) covered with alumina and a moving ground electrode. Ar was used as carrier gas, passing through a bubbler containing GMA. The plasma was ignited at 10 KHz in continuous and pulsed mode. When the discharge is pulsed, the off-time deposition can proceed through conventional polymerization reactions, thus a higher retention of functional groups in the coating can be achieved. In pulsed mode, the plasma-on time was fixed at 10 ms, while the off-time was in the range 10–80 ms. In the FTIR spectra reported in Figure 3 (top) it can be observed that coatings deposited in pulsed mode display less broad bands in the absorption regions of epoxy groups, indicating a higher retention of such groups from the monomer. Pulsing the discharge also affects the surface morphology, as highlighted by the SEM images in Figure 4 (bottom). It has been found that pulsed conditions, at the lowest duty cycle investigated (10 ms on, 80 ms off), lead to the fast deposition of adherent and smooth layers with the highest epoxy surface density, according to FTIR and XPS (18% of the total atomic carbon) data. PE-CVD GMA coatings were used to bind DspB to stainless-steel at ambient temperature; the immobilization was controlled by means of FTIR spectroscopy and then tested against biofilm forming *S. epidermidis* ATCC35984.

The antibiofilm activity onto coated and uncoated stainless-steel substrates is reported in Table 2 as percent of reduction of the adherent bacterial population. With respect to steel substrates uncoated or coated just with PE-CVD GMA, it can be seen that Dispersine-immobilized samples led to about 80% reduction of bacteria, indicating the efficacy of the immobilization of Dispersine in its active form on the coating. Similar GMA coated surfaces were also used to bind Laccase, a hydrolase enzyme, whose activity was confirmed in the degradation of a sulfamidic antibiotic [69].

## 4. Plasma Deposited Composite Coatings Embedding Organic Antibacterial Agents

When the prevention of bacterial colonization is required for short time (days–weeks), or for an entire body district and not only at the tissue/device interphase, bioactive coatings are demanded to release antibacterial drug in the surrounding medium in time- or stimuli-controlled way. Organic antibacterial compounds can be embedded in a composite coating by direct plasma deposition. The antibacterial agent can be a biomolecule with known antimicrobial activity such as lysozyme or nisin, or a synthetic drug like vancomicyn, or a commonly used quaternary ammonium salt. Essential natural oils can also be utilized [78]. The matrix can be properly chosen in order to match specific surface characteristics of the final product, e.g., biocompatibility, swelling, water resistance and so on. The drug release rate should also be controlled.

A recent approach to one-step PE-CVD of such nano/bio-composite coatings has been developed, for which a drug solution/suspension is injected in form of aerosol into a DBD through an atomizer, as shown in Figure 4 (bottom), while a polymerizable precursor is also fed. This approach is versatile, since the nature of the matrix and of the coupled biomolecule and their relative amount can be properly tuned to meet specific requirements. According to this approach, the precursor is fragmented in the discharge and plasma polymerized to form the matrix, while the active agent is simultaneously embedded within the growing film [79,80,81]. Damage to the biomolecule is limited by the milder fragmentation conditions typical of AP with respect to LP discharges, and by a thin protective solvent shell around the biomolecule, which can be effectively included in the coating without loss of structure and activity [80]. This concept has been proven with the optimization of Lysozyme (Lyz) containing antibacterial nano/bio-composite coatings. Ethylene was chosen as the precursor to form a hydrocarbon matrix, while Lyz was injected as a water solution aerosol [82].

The DBD reactor, schematized in Figure 4, consists of two parallel silver electrodes covered with 0.63 mm thick alumina plates (8 × 13 cm^2^; 5 mm gap). The feed is pumped through the discharge by an aspirator placed on the opposite side of the flow injection. The electrode setup is confined in a sealed Plexiglas^®^ chamber [83]. A flow rate of 2–5 slm of He passes through the atomizer (mod. 3076, TSI) to generate the Lyz water solution aerosol; 10 sccm ethylene were added to an auxiliary He inlet to keep the total flow rate at 5 slm. Crystalline polished p-doped Silicon substrates were used. A sinusoidal AC voltage was applied (6 KV_pp_, 4 KHz) at a power density of 0.25 W·cm^−2^.

**Figure 5:**
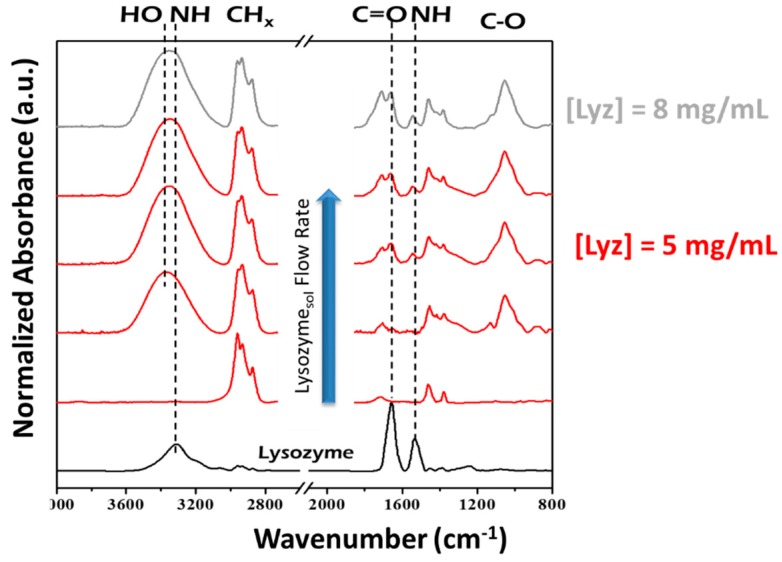
FT-IR spectra of: casted Lyz (black line); films deposited at atomizer flow rate of 0–5 slm with a 5 mg/mL Lyz solution (red curves); and at 5 slm atomizer flow rate with a 8 mg/mL Lyz solution (grey line). Adapted from [82], with permission from © 2015 John Wiley and Sons.

In Figure 5, the absorption FTIR spectra of films deposited at various atomizer He flow rates are reported, along with the spectrum of pure lysozyme. All coatings exhibit hydrocarbon absorption bands (3015–2777 cm^−1^, 1462 cm^−1^ and 1383 cm^−1^). The addition of the Lyz solution results in the appearance of typical amide bands (C=O stretch at 1660 cm^−1^, and NH bending at 1537 cm^−1^), consistent with the presence of protein or amino acid residues in the coating. The broad band at around 3350 cm^−1^ is due to the overlap of –OH and amidic –NH*_x_*. XPS analysis confirmed the presence of nitrogen in coatings prepared with the atomized Lyz solution. A deposition rate up to 75 nm/min was achieved for the coating with the highest Lyz content.

Matrix-Assisted Laser Desorption/Ionization Time-Of-Flight (MALDI-TOF, Micromass M@LDITM—LR, Waters MS Technologies, Manchester, UK; equipped with a nitrogen UV laser 337 nm) analysis was carried out to detect and identify lysozyme. The MALDI spectra of native Lyz and of Lyz-embedded coatings were similar, only showing the mono- and bi-charged ions of lysozyme at m/z 14,300 and 7150, respectively, indicating that the protein was still intact in the coating after the deposition [82].

The ability of the coatings, uniformly deposited onto 2.4 × 2.4 cm^2^ silicon shards, to release lysozyme was tested in distilled water by Reverse Phase-HPLC. The chromatogram of the solution obtained from the coatings was characterized by only one signal at the same elution time of native Lyz. No protein fractions could be detected, confirming MALDI-TOF data about the absence of main alterations of Lyz. On the other side, HPLC revealed that, for the coating deposited with atomized 8 mg/mL Lyz solution, after 1 h of incubation a Lyz concentration of 28 µg/mL was found in incubation water. Further immersion led to no more release, and the FT-IR analysis of the coating revealed that all loaded Lyz was released. In order to slow down the release, samples were prepared with a 50 nm hydrocarbon barrier layer on top of the nano/bio-composite coating, deposited in the same experimental conditions, from a He 5 slm/C_2_H_4_ 10 sccm feed (no Lyz aerosol). The added barrier layer consistently reduced the release, as reported in Table 3. This strategy is clearly valuable for designing thin systems with programmed release rate. The nano/bio-composite coating with a 50 nm barrier layer could release Lyz for 7 days of immersion in water.

The bioactivity of released lysozyme was tested with an agar diffusion test against *Micrococcus lisodeikticus*. When lysozyme diffused through the agar loaded with bacteria, agar turned from opaque to transparent, attesting for the antibacterial activity of the protein. Lysis net halos diameters are reported in Table 4. It can be observed that the solution released from the PE-CVD Lyz coating induced a halo with a diameter of about 8 mm, quite close to that originated from a 30 µg/mL standard protein solution. As expected, no halos were found for extract solutions of Lyz-free coatings. Such findings show the possibility to effectively include anti-microbial agents in PE-CVD coatings. Since the bio-activity seems to be retained, important applications can be envisioned for the dry manufacturing of drug carrier systems with programmed delivery.

## 5. Plasma Deposited Composite Coatings Embedding Inorganic Antibacterial Agents 

Certain metals are known to possess bactericidal properties, among them silver, titanium dioxide (TiO_2_), zinc oxide (ZnO) and copper oxide (CuO) have enabled the development of a new generation of biocides. Depending on the metal property, the biocide behavior of metals and metals oxides can be triggered by different mechanisms including the involvement of metals as catalytic co-factors in the generation of reactive oxygen species (ROS) [84]. In this mechanism, metals are involved both in cell oxidative stress and in triggering pro-inflammatory signal cascades that promote programmed cell death [85]. Another possible mechanism of action is based on the binding of the metals to atoms (e.g., O, N, and S), of donor ligands through strong and selective interactions that inhibit vital biological roles covered by the donors [86]. Metals and metal oxides can exert their function either in the cell membrane or in the intracellular region [87,88]. Generally, the biocidal activity of such inorganic antimicrobials has been found to be associated to the ionic form [89].

The antibacterial activity of silver and silver ions in proper concentrations, accompanied by low toxicity to human cells, is well known since a long time ago [90]; in addition, bacterial resistance is not developed. Silver has been incorporated into the surface of a variety of medical devices, such as vascular, urinary and peritoneal catheters, vascular grafts, prosthetic heart valve sewing rings, surgical sutures and fracture-fixation devices [91]. During the last years, nanotechnology has produced a new route to take advantage of the antimicrobial behavior of metals by synthesizing highly active metal nanoparticles (NP) [92]. 

**Figure 6:**
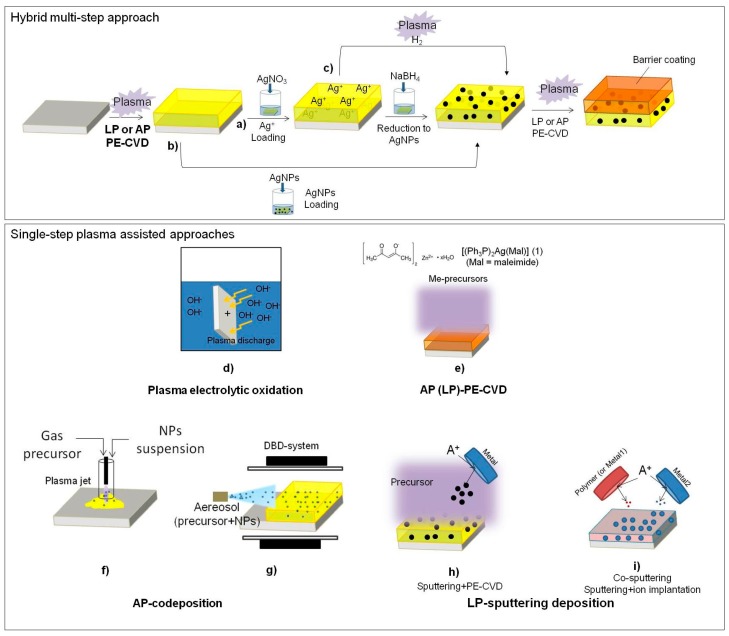
Scheme of plasma assisted approaches. The first route is a hybrid multi-step approach consisting of a PE-CVD coating containing carboxylic or amino groups that enhances (**a**) the adsorption of Ag^+^ ions or (**b**) of AgNPs at the surface and/or inside the coating. The reduction of Ag^+^ ions with NaBH_4_ (or trisodium citrate) or (**c**) through a plasma fed with H_2_ occurs. Finally, a PE-CVD barrier coating allows the control of silver release. The second group of processes are single step approaches totally based on plasma processes; (**d**) Electrolytic anodization is performed at high potential to ignite a discharge at material/liquid interface to change the chemical state of the surface and embedd antibacterial components (e.g., AgNO_3_) within its surface; (**e**) PE-CVD of a metal-containing volatile precursor (e.g., zinc acetate or silver bisphosphine, or maleimide complex) can be used to deposit metal-containing nanocomposite coatings; a plasma co-deposition of NPs and of an organic precursor allow to obtain a nanocomposite coating both in case of (**f**) an AP Plasma Jet or of (**g**) a DBD fed with an aereosol of a suspension of NPs; (**h**) A process based on plasma sputtering of a metal of interest and PE-CVD from a precursor is aimed at obtaining a metal-containing nanocomposite coating; (**i**) A contemporary sputtering of a polymer target and a metal one, or a deposition of the polymer of interest by PE-CVD and a contemporary ion implantation can lead to a thin layer containing nanoclusters of the metal of interest dispersed in an organic (or inorganic) matrix. Abbreviations: Mal: maleimide; Ph: phosphine.

Several surface modification approaches aimed at introducing inorganic antibacterial elements as well as NPs have been described so far, and comprehensive reviews have been published [8,20,93]: a scheme of the possible strategies for plasma-assisted approaches used to synthesize coatings containing antibacterial inorganic elements is shown in Figure 6. 

One of the plasma-assisted strategies, illustrated in Figure 6a–c, requires the plasma activation of the material surface and further exposure of the material to a solution of metal compounds or of NPs. The plasma process is used to improve the interaction between the metal (or NPs) and the surface, or for the further modification of the adsorbed metal (i.e., chemical reduction, and production of NPs). Leys et al. [94,95] described a three-step approach (Figure 6b) consisting of the AP PE-CVD of a silicone-like thin film using an AP plasma jet, followed by the dipping-adsorption of silver nano-particles (AgNPs), then by a final AP-PE-CVD of a silicone-like barrier layer that allowed the reduction and control of the release of Ag^+^ ions. Such coatings show antimicrobial activity against *P. auruginosa*, *S.* a*ureus*, and *C. albicans.* A *s*imilar strategy was performed also on nylon plasma-treated with a DBD in atmospheric air [96].

Hydrogel-like plasma deposited coatings (Figure 6a) have been used to fix Ag^+^ ions from an AgNO_3_ solution. The authors demonstrated control over the release of Ag^+^ by depositing a thin (<20 nm) plasma-polymer overlayer [97]. Following this strategy, some research groups proposed the PE-CVD of coatings from a proper organic precursor (i.e., acrylic acid or n-heptylamine) to absorb Ag^+^ ions from an AgNO_3_ solution and to reduce them with NaBH_4_ to form AgNPs embedded in the coating [98,99]. Such surfaces were found to be bioactive against *E. coli* and *S. aureus*.

**Figure 7:**
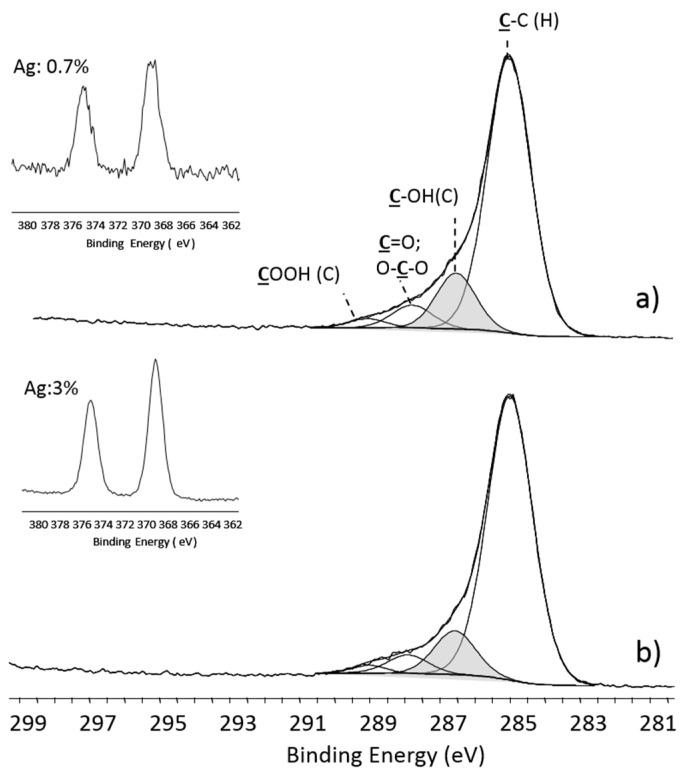
XPS spectra acquired on silver loaded PEO-like coatings after pre-conditioning of the coating at: (**a**) pH 4; and (**b**) pH 8.

Plasma-deposited PEO-like coatings exhibit hydrogel behavior in water [100]; for this reason, they have been investigated by the authors to absorb Ag^+^ ions to be reduced to metal silver. PEO-like coatings were plasma-deposited at LP [101] at 60 W power (high fragmentation conditions), 20 sccm Ar, 0.25 sccm DEGDME, 50 mTorr for 15 min. XPS show high cross-linking of the coating, attested by the low amount of -CH_2_CH_2_O- moieties (i.e., low PEO-character, grey peak in Figure 7), and by the presence of -COOH groups. These coatings were chosen to uptake Ag^+^ ions through ionic bonds with their ionized -COOH groups. Following the procedure of ref. [102], the samples were rinsed for 15 min in solutions at pH 4 (HCl 10^−4^ M) and pH 8 (NaOH 10^−6^ M), then they were exposed for 15 h to a 1 mg/mL solution of AgNO_3_ to absorb Ag^+^ ions, and finally immersed in a water solution of trisodium citrate (0.7 mg/mL) for 24 h for reducing Ag^+^ to Ag. As shown in Figure 7, the coatings conditioned at pH 8 were able to embed a higher amount of silver, likely due to the interactions of Ag^+^ with the surface carboxylate ions formed at higher extent than at pH 4. The concentration of silver detected is similar to that reported by Kumar et al. [99] who showed an abatement of bacterial concentration higher than 99.7% compared to untreated PET against *E. coli* and *S. aureus*. The advantage of such approach consists in the possibility to control the loading of silver by controlling the chemical composition of the deposited material and the pH; the disadvantage is that at least two steps are necessary to obtain such coatings. To overcome this limit, one can incorporate AgNPs into a PE-CVD coating by a simultaneous deposition of a metal (metal oxide) and a plasma organic or inorganic matrix (Figure 6d–i). There are at least three approaches, uniquely based on plasma processing, to perform such depositions: (1) plasma electrolytic oxidation (Figure 6d); (2) plasma deposition from a metal containing precursor (Figure 6e); and (3) dual strategies (Figure 6f–i).

A new surface modification approach involves the electrochemical conversion of metal surfaces (i.e., Titanium) into more desirable chemistries and topographies by using anodizing or plasma electrolytic oxidation processes (Figure 6d) [103,104]. This technique produces coatings characterized by an excellent adhesion to substrates; however, it requires the use of a metal substrate. To overcome this limit, hybrid processes where a plasma-assisted approach is coupled with another one are proposed. The most frequent examples of hybrid plasma strategies are reported in Figure 6e, PE-CVD from a precursor containing a complex of the metal of interest or organo-metal compound [105,106]; Figure 6f,g AP plasma co-deposition of an organic coating and AgNPs or AgNPs precursors [107,108,109]; and Figure 6h,i, simultaneous sputtering of a target coated with the antibacterial agent (i.e., Cu, Ag, TiO_2_) and PE-CVD of an organic precursor or ion beam deposition [110,111].

Polymer-like coatings represent a potentially interesting approach to controlled drug release of inorganic antimicrobial compounds. Plasma polymerization of an organic film while adding an antibacterial agent by sputtering from a target is one of the most widely used and well characterized approach in this field. Silicone-like/Ag [112], silicone-like/Cu, diamond-like/Ag, ethylene-like/Ag [113] and PEO-like/Ag nanocomposite coatings have been produced in this way. The formation of Ag clusters in different matrices is promoted by the high mobility of Ag atoms in the growing coating, which leads to aggregation of Ag clusters [114], whose size is strongly dependent on the Ag content [115]; often, the silver distribution is not uniform along the thickness of the coatings [116].

To deposit Ag/PEO-like nanocomposite coatings we used a LP deposition system with parallel asymmetrical plate electrodes. The smallest one (7 cm dia) covered with a 2 mm thick Ag lamina, is connected through a matching network to a RF (13.56 MHz) power supply, the other one (25 cm dia), sample holder, is grounded. The asymmetric configuration allows a strong positive ion-bombardment on the silver electrode, at proper pressure, power and gas composition, that sputters Ag atoms from the target. At the same time a PEO-like coating develops at the ground electrode, where the ion bombardment is reduced, that incorporate Ag atoms and nanometric clusters. DEGDME vapors were flown at 0.25 sccm in all experiments, mixed with a variable flow of Ar (10–20 sccm). The input power was changed from 10 to 60 W at constant pressure (50 mTorr) to vary the amount of sputtered silver. In these conditions, the deposition of a coating at the silver electrode, that would block the sputtering of Ag atoms, is prevented. By adjusting the process parameters, the coatings could be tailored (e.g., Ag content, dimensions distribution and morphology of Ag clusters) to different requirements for specific applications, where they act as Ag reservoirs. Bacterial adhesion on such samples was evaluated both in static and under flow conditions in order to investigate the combined effect of flow and surface chemistry on the ica gene expression [117]. By a comparison between Ag/PEO-like coatings and highly cross-linked PEO-like ones, but with no silver embedded, prepared in the same conditions, only 30% of adherent bacteria on Ag/PEO-like coatings remained alive. It is known that assessment of icaA DBC operon gene expression is crucial to the understanding of the pathogenesis of *S. epidermidis* infections [118]. Higher expressions of icaA genes were observed for bacteria in contact with Ag/PEO-like coatings with respect to those on the corresponding highly cross-linked PEO-like ones with no silver.

**Figure 8:**
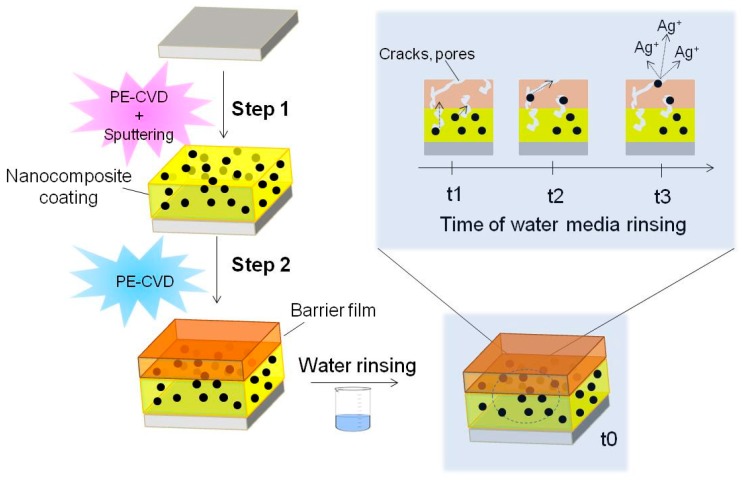
The scheme of silver ions generation and diffusion into a liquid medium during time starting from soon after the plasma processes. In the scheme, the first layer acts as the reservation layer while the outermost layer is used to control diffusion of silver through the coating. The generation of silver ions pass through the oxidation of silver when is in the elemental state [119].

In order to preserve the antibacterial efficiency while protecting eukaryotic cells from the toxic effect of the excessive Ag^+^ release, a barrier layer was deposited on top of plasma deposited Ag/PEO-like coatings [119] to limit the release of Ag^+^ ions in water media (Figure 8). Thickness and chemical composition of the barrier significantly influence the release that may occur through small cracks and pores, as well as by diffusion through the coating and the barrier together, slightly swollen in water media [98,109]. The barrier layer can control and prolong the antibacterial effect.

We have tested the Ag^+^ release in cell culture Dulbecco’s Modified Eagle Medium (DMEM) during eukaryotic cell culture experiments, a very complex environment were the release could be altered by protein adsorption and damage of the coating induced by adhering cells. After 24, 48 and 72 h of culture, 500 µL of medium were digested in 2 mL of HNO_3_ (70%, Sigma Aldrich, St. Louis, MO, USA) to oxidize the organic matter, then dissolved in 2% *v*/*v* HNO_3_ solution in water. The amount of silver released was measured with ICP Atomic Spectroscopy. Ag/PEO-like coatings containing 5% of silver, as detected by XPS, were coated with 15 nm and 60 nm thick PEO-like barrier coatings with 20% PEO-character. An abatement of the released silver resulted with the 60 nm barrier, at all incubation times investigated, while no effect was observed with the 15 nm barrier. At 24 h the amount of released silver from samples coated with 60 nm overlayers was 0.5 ± 0.02 ppm∙cm^−2^, after 72 h the same coatings released 1.5 ± 0.2 ppm∙cm^−2^ while the release was up to 5 ± 0.5 ppm∙cm^−2^ without the barrier. When at least 1 cm^2^ samples were used, independently on the barrier, the amount of released silver was higher than the MIC of Ag^+^ ions (0.9–1.7 ppm) for most gram negative and positive bacteria [120].

**Figure 9:**
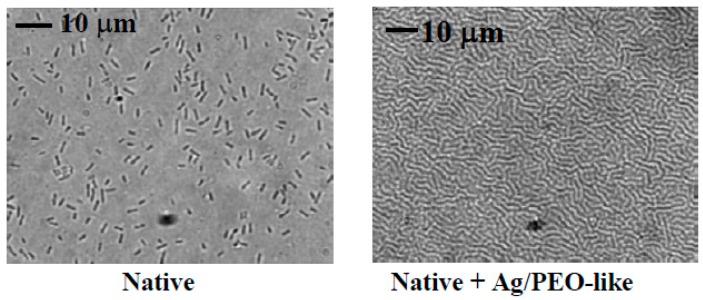
Micrographs of *P. aeuruginosa* adhesion onto native PVC Endotracheal Tubes as well (native) and coated by Ag/PEO-like coatings containing 3% of silver. The mosaic pattern seen on the Ag/PEO-like micrograph is due to the wrinkling of the underlying PVC substrate, adapted from [101], with permission of © 2005 John Wiley and Sons.

Ag/PEO-like coatings prepared by us containing from 1% [101] to 25% of silver [101,117], show antibacterial effects against *P. aeuruginosa*, *S. epidermis*, and *E. coli*; coatings with 3% silver showed a 100% reduction of *P. aeuruginosa* adhesion, as reported in Figure 9.

**Figure 10:**
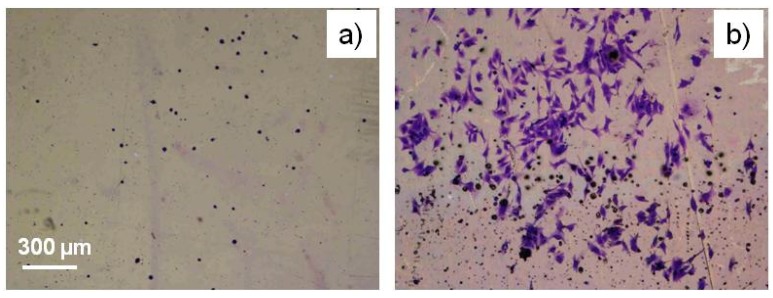
SAOS 2 Coomassie Blue stained cells on Ag/PEO-like (5% Ag) surfaces uncoated (**a**) and coated (**b**) with a 60 nm thick barrier coating (PEO character 20%).

To check the toxicity of Ag/PEO-like coatings for eukaryotic cells, in vitro cito-toxicity tests were performed with SAOS2 cells grown on coatings loaded with 5% of silver, with and without barrier layers. Coatings without the barrier resulted somewhat toxic, and could promote the adhesion of only few round shaped cells, due to the high release of Ag^+^ but, maybe, also to the direct contact of cells with Ag clusters protruding from the surface. On samples with a 60 nm thick barrier coating (20% PEO character, the same of the matrix of the Ag/PEO material), instead, more cells could adhere and spread, as shown in Figure 10, indicating a healthier environment for the cells due to the lowered release of Ag^+^.

## 6. Conclusions and Perspectives

An overview on plasma-based technologies optimized for the deposition of antimicrobial coatings has been given in this paper, where most of the surfaces described have been developed in our laboratory. In particular, we believe to have shown the power and the flexibility of LP and AP plasma-based techniques in engineering antibacterial coatings for the prevention of infections in biomedical materials, with approaches that could probably also be exploited in other fields, such as active food packaging, marine fouling and industrial food processing, where bacterial attachment and contamination, and biofilm formation are of concern.

Plasma Technology, indeed, is nowadays expressing several newer approaches to surface modification, decontamination/sterilization methods and medical therapies in several fields where it was not considered before, where of bacteria proliferation always has to be controlled, e.g., in Medicine for wound healing and cancer treatments [121], and in Agriculture for seed germination and food decontamination [122].

Among several approaches presented, in particular, the aerosol-assisted AP plasma synthesis of nano/bio-composite coatings with antibacterial molecules embedded in, and releasable from, plasma-deposited nanometric coatings, possibly implemented by plasma-deposited diffusive layers is considered, in perspective, among the most promising approaches to antibacterial surfaces and to drug-release systems in general. We believe that this synthetic method will be strongly implemented, in the near future, with the increased use of natural molecules, e.g., available from extracts and oils from plants, flowers and fruits, for an ecologic approach to antibacterial material and surfaces.

## Figures and Tables

**Table 1:** Examples of bioactive surfaces with antibacterial properties.

**Drug Delivery Systems**			
**Technology**	**Antibacterial Agent**	**Bacterial Target**	**Ref.**
Drop Casting/Dip Coating	Chlorexidin	*-*	[14]
	Immunoglobulin G	*Escherichia coli*	[15]
Layer-by-Layer	Defensin	*Micrococcus luteus* *E. coli*	[16]
	PEGylated polylysine	*E. coli*	[18]
	Triclosan	*Staphylococcus aureus*	[19]
Sol-gel	Ag^+^/Zn^2+^	*Staphylococcus mutans*	[28]
	CuO	*S. aureus* *E. coli*	[29]
Electrochemical Deposition	Cu^2+^	*S. aureus* *E. coli*	[11]
	Ag^+^	*S. aureus* *Pseudomonas. Aeruginosa* *E. coli*	[12]
	Collagen	*-*	[22]
	Penicillin/Streptomicin	*-*	[23]
**Covalent Immobilization**			
**Surface Reactive groups**	**Antibacterial Ingredient**	**Bacterial target**	**Ref. **
-COOH/-F	Melimine	*P. aeruginosa* *S. aureus*	[25]
-NH_2_	Algnic acid	*-*	[26]
-COCl	Vancomycin	*S. aureus*	[30]

**Table 2:** Anti-biofouling activity against *S. epidermidis* of modified stainless-steel surfaces compared to uncoated ones. Adapted from ref. [69], with permission from © 2015 John Wiley and Sons.

Samples	Reduction of Adherent Population [%]
Stainless steel	0
ppGMA layers	0
Dsp B immobilized at pH 8.5	84 ± 11

**Table 3:** Released Lysozyme from composite coatings deposited on 2.4 × 2.4 cm silicon shards at an atomizer flow rate of 5 slm with a 8 mg/mL Lyz solution, without and with a 50 nm hydrocarbon PE-CVD barrier layer.

**Released Lysozyme [µg/mL]**		**15 Min**	**1 Day**	**7 Days**
	Without barrier coating	20	28	-
	With barrier coating	2	15	18

**Table 4:** Agar diffusion activity test results for the Lyz-containing coating from a composite layer prepared with a 8 mg/mL solution aerosol, at 5 slm of He. Based on [82].

Sample	Inhibition Halo Diameter [mm]
C_2_H_4_/Lyz_sol_ coating	8 ± 1
C_2_H_4_/H_2_O plasma deposited coating (control)	0
Lyz std solution (10 µg/mL)	0
Lyz std solution (30 µg/mL)	6 ± 1
Lyz std solution (300 µg/mL)	12 ± 1
Blank (negative control)	0
